# Real-Time Sound Source Localization Based on the Ground-Reflection Compensation Algorithm

**DOI:** 10.3390/s26185685

**Published:** 2026-09-08

**Authors:** Shengkai Zhao, Zhen Huang, Zhiwen Ma, Zhengyang Zhang, Zhenxin He, Hongjie Cheng

**Affiliations:** School of Missile Engineering, Rocket Force University of Engineering, Xi’an 710025, China

**Keywords:** sound source localization, ground-reflection compensation, MEMS array, maximum likelihood estimation

## Abstract

Sound source localization based on microphone arrays has attracted extensive research interest in the fields of speech communication, detection and tracking. However, conventional near-field beamforming typically assumes free-field propagation and ignores coherent ground reflections, which bias phase estimates at floor-mounted arrays and degrade localization accuracy. To address this, the total Green’s function is reformulated by embedding the image-source reflected path directly into the beamforming propagation model. Unlike conventional approaches that assume free-field propagation, this formulation enables phase compensation to jointly account for the direct and ground-reflected wavefronts. The in-phase superposition of signals is then realized through frequency-domain phase compensation, and the sound source position is estimated via the maximum likelihood criterion. Subsequently, a dual-mode operation mechanism of automatic broadband scanning and manual single-frequency analysis is implemented, with frequency-weighted wideband spatial spectrum processing for noise and aliasing suppression. Finally, a real-time sound source localization system based on a 4 × 4 MEMS array is designed. Experimental results show that sound source localization in a broad frequency band can be achieved at standoff distances of 0.1 m–0.3 m from the array plane, with a localization accuracy of less than 0.015 m. The proposed method demonstrates strong robustness against ground-reflection interference and has potential applications in acoustic monitoring, industrial fault diagnosis and other related areas.

## 1. Introduction

Sound source localization technology, as a core research direction in the field of acoustic signal processing, collects acoustic signals via microphone arrays and accurately estimates the sound source position. It has broad applications in noise pollution control, industrial equipment fault diagnosis, human–computer interaction, and robot navigation [1]. With the upgrading of industrial intellectualization and the growing demand for acoustic technology in people’s livelihoods, this technology is confronted with greater challenges in terms of localization accuracy, environmental adaptability, real-time performance and engineering practicability. These challenges have prompted domestic and foreign scholars to conduct a series of studies on core directions such as array configuration optimization, algorithm improvement and scene adaptation [2,3,4].

Current research has achieved phased results, which are mainly divided into three categories: array configuration optimization, specific scene adaptation, and localization algorithm improvement. Each category addresses a distinct aspect of the localization problem, but also exhibits inherent limitations when applied in isolation. In terms of array configuration, scholars have compared the performance of various two-dimensional arrays such as BK, Ring and starfish arrays, proving that the starfish configuration is the optimal one [5]. Meanwhile, 3D/large-scale configurations including 64-element spherical arrays and nested arrays have been developed, which have clarified the law of influence of installation deviation on localization accuracy [6,7] and emphasized the engineering significance of optimal structural design [8]. Furthermore, aiming at the drawback that 3D configurations exhibit poor performance at high Helmholtz numbers due to large spatial and spectral deviations, it is proposed to adopt a stellated octahedron shape, which can standardize the directional response of the array while maintaining a small number of microphones [9,10]. In the selection of microphones, targeting the problem that condenser microphones adopted in traditional microphone arrays are susceptible to electromagnetic interference, a solution involving replacing them with fiber optic acoustic sensors is put forward; further, the latter exhibit higher sensitivity and accuracy than traditional microphones [11]. While these optimized configurations improve spatial resolution and suppress sidelobes, they do not address propagation-model mismatch: regardless of array geometry, the beamforming kernel typically remains based on a free-field Green’s function and is therefore vulnerable to coherent reflections from the ground or equipment surfaces. In the aspect of scene adaptation, relevant research targets addressing the demands of different fields: in industrial scenarios, the fault diagnosis of components such as gears and pipelines has been realized, and a reference for the selection of near–far-field models has been provided [12,13]; for the noise source visualization scenario, optimized dipole localization combined with the rotary array algorithm is adopted, thereby improving the localization accuracy of the algorithm [14,15], and the Time Difference of Arrival (TDOA) is calculated via the generalized cross-correlation algorithm to improve the anti-interference performance of time delay estimation in specific scenarios [16]. Meanwhile, addressing the problem of insufficient datasets for machine learning methods, scholars have adopted a data generation framework for parallel computing on multiple machines, which accelerates the dataset generation process [17]. In terms of algorithm improvement, continuous optimizations have been made to the traditional Time Difference of Arrival (TDOA) and beamforming algorithms. For instance, the traditional beamforming method is combined with Ensemble Empirical Mode Decomposition (EEMD) and the Estimation of Signal Parameters via Rotational Invariance Techniques (ESPRIT) to construct a beamformer, which is applied to resolve aliasing and distortion in acoustic signals [12]. To address the problem of low robustness under low signal-to-noise ratio (SNR) conditions, Tang et al. [18] proposed an incremental broad learning system (a fast incremental neural network that realizes low-complexity online learning through random feature mapping) to estimate the sound source position. Instead of the traditional grid-based method, scholar Jun Tang adopted the Fibonacci Sphere Algorithm (FSA) to ensure the uniform distribution of microphone array elements, and combined it with a time-domain neural network to optimize the sound source localization algorithm. This method can directly predict the target coordinates and circumvent the inherent spatial resolution limitation of grid-based techniques, but it has high computational complexity and poor real-time performance [19]. The deep learning methods represented by his research can effectively improve adaptability to complex environments; among such methods, models including Deep Neural Networks (DNNs), incremental broad learning and lightweight fully convolutional neural networks demonstrate higher localization accuracy under low signal-to-noise ratio (SNR) and strong reverberation scenarios [20,21]. These scene-specific methods are effective within their target scenarios, but they share a common reliance on the free-field or anechoic assumption; their performance degrades when reflective surfaces are present near the array, a condition common in industrial floor-mounted deployments. Among all these algorithms, beamforming algorithms, especially those represented by the maximum likelihood estimation, are more suitable for sound source localization and broadband acoustic signal processing in engineering applications due to their advantages of low computational complexity and easy implementation. However, traditional beamforming algorithms, which are most widely used in engineering, generally adopt the free-field assumption and ignore the reflection effect of the ground or equipment surface [22,23]. This leads to the superposition of direct waves and reflected waves, resulting in serious positioning deviation. In addition, most existing algorithms adopt a dense grid search strategy without optimization, which leads to high computational complexity and cannot meet the real-time requirements of industrial online monitoring. Although deep learning methods have achieved good results in low SNR and strong reverberation scenarios [24,25], they rely on large-scale datasets and have poor generalization ability in unknown environments. In summary, none of the three categories above directly addresses the gap between the free-field assumption of conventional beamforming and the ground-reflection reality encountered in floor-mounted array deployments, which motivates the present work.

To address this limitation, we propose a method that embeds ground-reflection compensation directly into the beamforming Green’s function. Specifically, the total Green’s function is reformulated to jointly model the direct path and the image-source reflected path, so that the subsequent phase compensation operates on the combined acoustic field rather than on the direct path alone. This integration converts ground reflection from a source of coherent interference into a constructive contribution at the true source position. Compared with existing methods, MUSIC is narrowband and model-sensitive, SRP-PHAT is costly for real-time broadband search, adaptive filters require a reference signal, and existing reflection compensation typically operates as a separate stage. The documented limitations of these methods motivate the design choices of this work: MUSIC assumes narrowband or tonal sources, limiting its applicability to broadband signals; SRP-PHAT is computationally demanding for real-time operation; adaptive filtering requires a reference signal. Section 4 presents a quantitative comparison of representative methods from the literature against the proposed approach. The proposed method differs by embedding compensation into the beamforming kernel for joint single-step processing. This integration reduces the localization RMSE from 4.2 cm to 0.9 cm, surpassing all experimental benchmarks in Section 4 by a factor of 3 to 32. This paper addresses three research questions: how to compensate coherent ground reflections within the beamforming framework itself (RQ1, Section 2); to what extent this integration improves accuracy and sidelobe suppression over free-field beamforming (RQ2, Section 4); and whether real-time operation is achievable on a 4 × 4 MEMS array (RQ3, Section 3 and Section 4). The method is implemented on a 4 × 4 MEMS array: comparative experiments show that the localization RMSE is reduced from 4.2 cm to 0.9 cm, and the main-to-sidelobe peak ratio improves from 10.5 dB to 19.3 dB. A complete list of symbols is provided in Nomenclature.

## 2. Methodology

### 2.1. Free-Field Sound Source Modeling and Ground-Reflection Compensation Based on Green’s Function

Actual sound source localization scenarios often involve the ground-reflection effect; thus, it is necessary to introduce a compensation mechanism on the basis of free-field modeling. Based on the assumption of a homogeneous ideal fluid medium, this paper starts from the wave equation, derives the frequency-domain Helmholtz equation via Fourier transform, and solves for the free-space Green’s function to characterize the sound field distribution of a point sound source [26]. Finally, the method of images is adopted to realize the boundary condition compensation for ground reflection, thereby establishing a sound field model that is close to actual application scenarios [27].

In a homogeneous, lossless and ideal fluid medium, the propagation of small-amplitude acoustic waves satisfies the linear wave equation under the assumptions of linear propagation, no viscosity, no heat conduction, and small-amplitude disturbance. The time-domain form of the wave equation is given by

(1) $$\nabla^{2}p\left(r,t\right)-\frac{1}{c^{2}}\frac{\partial^{2}p\left(r,t\right)}{\partial t^{2}}=0$$

In Equation (1), $\nabla^{2}=\frac{\partial^{2}}{\partial x^{2}}+\frac{\partial^{2}}{\partial y^{2}}+\frac{\partial^{2}}{\partial z^{2}}$ denotes the Laplacian operator, p(r,t) represents the time-domain sound pressure at the spatial coordinate r=(x,y,z) and time t, and c=343 m/s is the sound velocity under standard atmospheric conditions.

To analyze the characteristics of the frequency-domain sound field, the temporal Fourier transform is applied to both sides of Equation (1). According to the differential property of the Fourier transform,

(2) $$F\left[\frac{\partial^{2}p}{\partial t^{2}}\right]=(j\omega {)}^{2}P(r,\omega )=-\omega^{2}P\left(r,\omega \right)$$

where ω=2πf is the angular frequency, f is the acoustic wave frequency, and $P(r,\omega )=F\left[p(r,t)\right]$ denotes the frequency-domain sound pressure. Substituting this into the wave equation and rearranging the terms, the frequency-domain Helmholtz equation is obtained as follows:

(3) $$\nabla^{2}P\left(r,\omega \right)+k^{2}P\left(r,\omega \right)=0$$

In the equation, k is the wavenumber, which characterizes the spatial frequency characteristic of the acoustic wave, and its physical meaning is the phase variation of the acoustic wave per unit length.

For a point sound source located at the coordinate $r_{0}=(x_{0},y_{0},z_{0})$, it is necessary to introduce the Dirac delta function $\;\delta (r-r_{0})$ to describe the source term distribution of the concentrated sound source. In this case, the inhomogeneous form of the Helmholtz equation is given by

(4) $$\nabla^{2}G\left(r,r_{0},\omega \right)+k^{2}G\left(r,r_{0},\omega \right)=-\delta \left(r-r_{0}\right)$$

In the equation, $G(r,r_{0},\omega )$ is precisely the free-space Green′s function, whose physical meaning is the frequency-domain complex sound pressure generated at the spatial position r by a point sound source with unit intensity, and it is used to quantitatively characterize the sound field propagation law of a point sound source.

Considering the spherical symmetry of the sound field generated by a point sound source, the spherical coordinate system (R,θ,ϕ) is adopted to simplify the solution (where $R=|r-r_{0}|$ denotes the Euclidean distance between the sound source and the observation point). In the spherical coordinate system, the Laplacian operator can be simplified to $\nabla^{2}=\frac{1}{R^{2}}\frac{\partial }{\partial R}\left(R^{2}\frac{\partial }{\partial R}\right)$, and the Green′s function is only a function of R, i.e., G=G(R). Substituting this into Equation (4), the equation reduces to a homogeneous form when R≠0 (far from the point sound source, where the source term is zero):

(5) $$\frac{1}{R^{2}}\frac{d}{dR}\left(R^{2}\frac{dG}{dR}\right)+k^{2}G=0$$

Let $G(R)=\frac{\psi (R)}{R}$ and substitute it into Equation (5); simplification yields the equation $\psi^{\prime \prime }(R)+k^{2}\psi (R)=0$, whose general solution is $\psi (R)=Ae^{jkR}+Be^{-jkR}$ (where A and B are undetermined coefficients). In combination with the radiation boundary condition (acoustic waves must radiate outward from the sound source rather than converge inward), the diverging wave solution with *B* = 0 is adopted, and the free-space Green’s function is finally obtained as

(6) $$G\left(r,r_{0},\omega \right)=\frac{1}{4\pi R}e^{-jkR}=\frac{1}{4\pi \left|r-r_{0}\right|}e^{-jk\left|r-r_{0}\right|}$$

In Equation (6), the denominator 4πR reflects the spherical spreading attenuation characteristic of spherical waves, and the molecular complex exponential term $e^{-jkR}$ characterizes the phase delay during acoustic wave propagation.

When the sound source and the microphone array are close to the ground, it is necessary to take into account the influence of ground reflection on the sound field. The image-source model assumes: (a) an infinite flat reflecting plane; (b) spatially uniform ground impedance; (c) pure specular reflection; (d) a real, frequency-independent reflection coefficient; and (e) a homogeneous, quiescent medium. According to the acoustic boundary conditions, the particle normal velocity at the rigid ground is zero, i.e., $\frac{\partial p(r,t)}{\partial n}{|}_{z\;=\;0}=0$ (the *z*-axis is taken to be perpendicular to the ground and pointing upward, where n denotes the ground normal direction). The method of images is adopted to satisfy this boundary condition, whose core idea is to replace the effect of ground reflection by constructing a virtual image sound source, such that the sound field in the original half-space (z ≥0) is equivalent to the superposition of the sound fields generated by the real sound source and the virtual image sound source in the full space.

**Figure 1:**
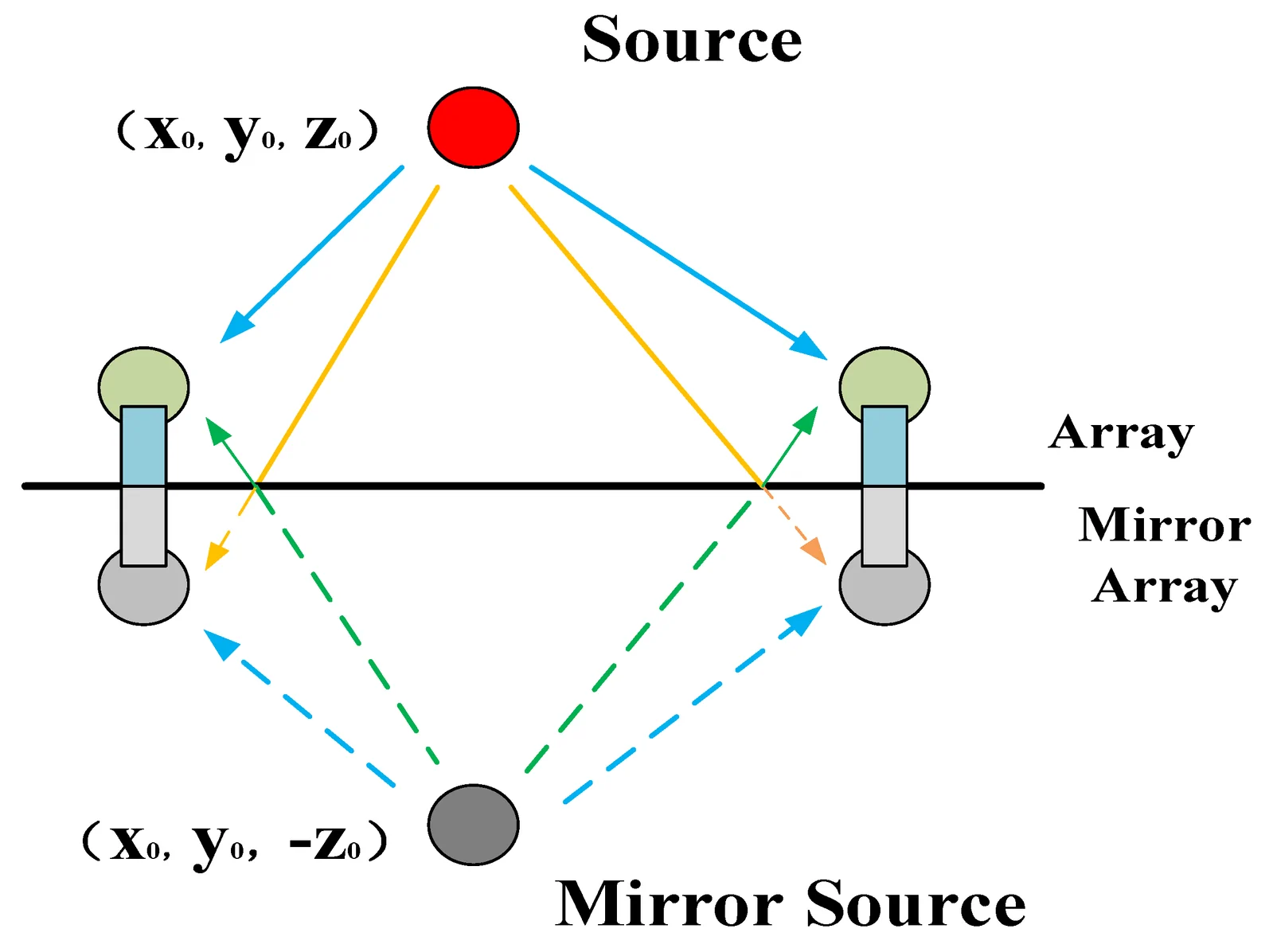
Diagram of direct and reflected wave superposition of acoustic signals.

As shown in Figure 1, let the position of the real sound source be $r_{0}=(x_{0},y_{0},z_{0})$ (at a height $z_{0}$ above the ground), and a virtual image sound source is constructed at the mirror-symmetric position $r_{0^{\prime }}=(x_{0},y_{0},-z_{0})$ with a surface-dependent reflection coefficient α, which may be complex-valued for absorptive surfaces. Typical |*α*|: concrete 0.93–0.98, asphalt 0.85–0.95, compacted soil 0.70–0.88, grassland 0.40–0.65, carpet 0.30–0.50. The present experiments use concrete (*α* ≈ 0.95); user-defined α is supported for other surfaces [28]. A real-valued α scales only the magnitude of the reflected term; a complex α would contribute an additional phase offset to the reflected path.

In this case, the total Green’s function considering ground reflection is the superposition of the Green’s function of the real sound source and the Green’s function of the virtual image sound source:

(7) $$G_{total}\left(r,r_{0},\omega \right)=G\left(r,r_{0},\omega \right)+\alpha \cdot G\left(r,r_{0^{\prime }},\omega \right)$$

Substituting the free-space Green’s function of Equation (6) into Equation (7) yields the explicit form of the total Green’s function, where the first term is the direct spherical wave from the true source at r_0_ and the second term is the reflected spherical wave from the image source at $r_{0^{\prime }}$. At the observation point $r_{0}$, the two contributions interfere with a phase difference $\Delta \varphi =k\left(\left|r-r_{0^{\prime }}|-|r-r_{0}\right|\right)$ governed by the path-length difference between the reflected and direct rays. The beamforming algorithm subsequently computes the model-predicted phase at each microphone and applies the inverse correction, so that the direct and reflected arrivals combine constructively at the true source position while canceling elsewhere. Accordingly, the total Green’s function is expressed as

(8) $$G_{total}\left(r,r_{0},\omega \right)=\frac{1}{4\pi \left|r-r_{0}\right|}e^{-jk\left|r-r_{0}\right|}+\alpha \cdot \frac{1}{4\pi \left|r-r_{0^{\prime }}\right|}e^{-jk\left|r-r_{0^{\prime }}\right|}$$

In the equation, $\left|r-r_{0^{\prime }}\right|$ denotes the Euclidean distance between the virtual image sound source and the observation point r.

After introducing the mirror compensation for sound source distribution, the system establishes a complete near-field acoustic propagation model that incorporates both direct sound and reflected sound. The core output of this physical model is the total propagation delay from each microphone position relative to the trial sound source point, which accurately characterizes the phase shift of the signal induced by path differences. In the process of phase-matched beamforming, the system takes this as the theoretical basis to perform inverse phase compensation on the actual signals received by each microphone channel, and the compensation amount is exactly equal to the phase shift corresponding to the delay predicted by the model.

In addition, it should be noted that in near-field beamforming, phase difference is the dominant factor determining sound source localization accuracy, while the influence of amplitude difference on localization results is far smaller than that. For the sound source localization algorithm in this paper, phase-only compensation is sufficient to achieve adequate localization accuracy, and it can significantly reduce computational complexity [29]. The compensation phase is the argument of the two-term sum in Equation (10), which jointly accounts for both paths.

### 2.2. Principle of Sound Source Localization Based on Phase Matching

**Figure 2:**
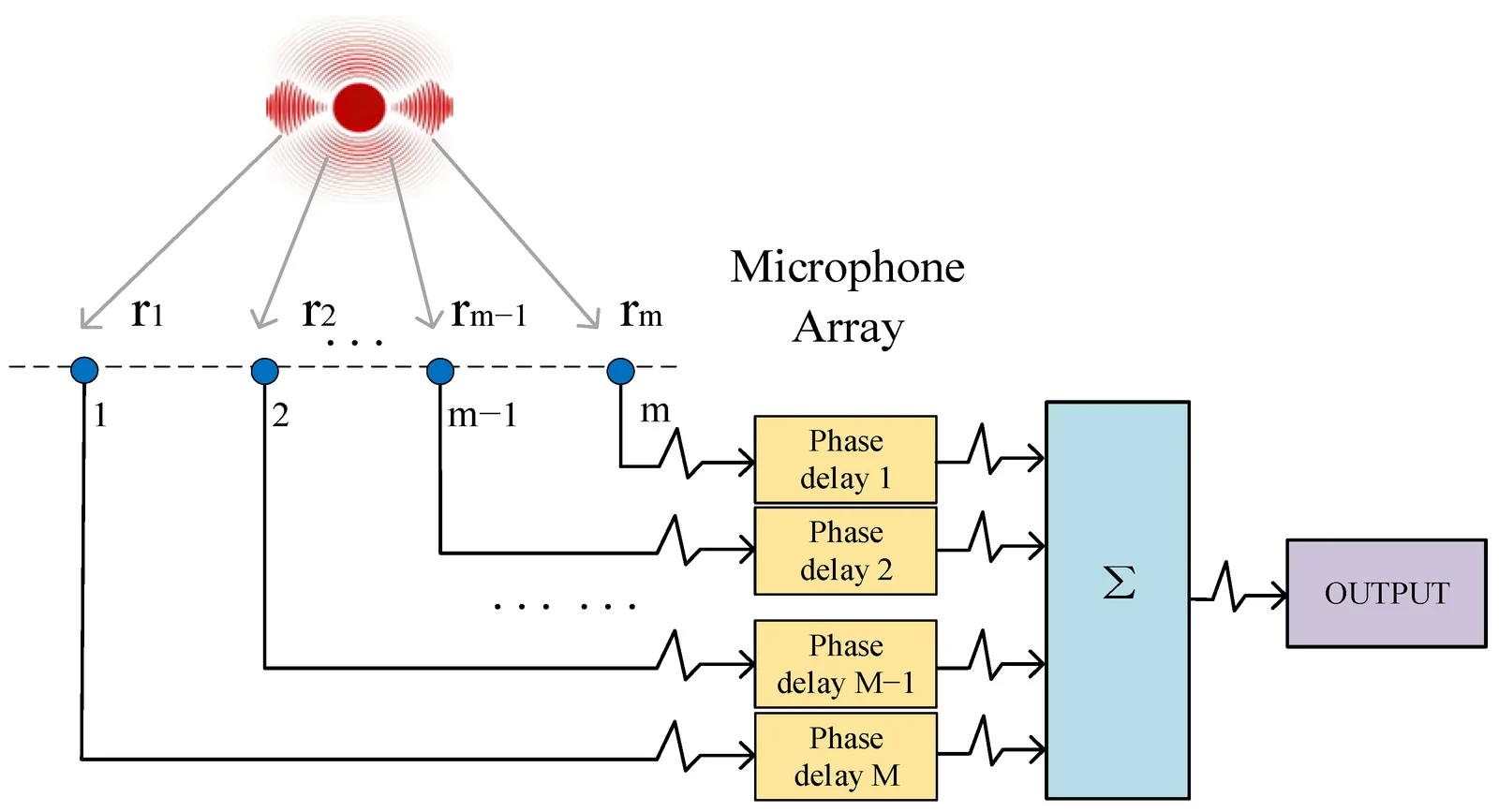
Schematic diagram of sound source localization based on microphone array.

Acoustic waves emitted by a sound source propagate in the form of spherical waves, generating path differences when reaching different microphones in the array and thus introducing phase differences, as shown in Figure 2. The sound source localization system compensates for this difference by configuring a corresponding phase delay module for the output signal of each microphone, enabling the signals from the target direction to achieve in-phase superposition in the summing unit. This enhances the target sound source signal while suppressing interferences and noise, and finally outputs the focused audio signal.

As shown in the figure, considering an array system composed of M microphones, in the frequency domain, the acoustic signal received by the m-th microphone can be expressed as

(9) $$X_{m}\left(\omega \right)=S\left(\omega \right)G_{m}\left(\omega ,p_{s}\right)+N_{m}\left(\omega \right),\;m=1,2,\ldots ,M$$

where S(ω) denotes the sound source signal spectrum, $p_{s}=[x_{s},y_{s},z_{s}{]}^{T}$ is the sound source position coordinate (with *T* representing the matrix transpose operation), and $N_{m}\left(\omega \right)$ is the additive noise. The propagation function $G_{m}(\omega ,p_{s})$ characterizes the propagation characteristics of acoustic waves from the source point to the m-th microphone. When ground reflection is taken into account, it can be decomposed into the sum of a direct component and a reflected component:

(10) $$G_{m}\left(\omega ,p_{s}\right)=\frac{e^{-j\omega \tau_{d,m}}}{4\pi r_{d,m}}+\alpha \frac{e^{-j\omega \tau_{r,m}}}{4\pi r_{r,m}}$$

In the equation, $r_{d,m}=\;∥p_{s}-p_{m}∥$ denotes the direct-path distance, $\tau_{d,m}=r_{d,m}/c$ is the corresponding time delay, α is the ground-reflection coefficient, and $r_{r,m}$ and $\tau_{r,m}$ are the distance and time delay of the reflected path, respectively.

The basic idea of beamforming is to apply phase compensation to the signals of each channel, enabling the signal components from a specific trial direction p to achieve in-phase superposition. Throughout this paper, bold symbols denote position vectors (*p*, $p_{s}$, $p_{alias,\;k}$, $p_{m}$); scalar quantities are written in regular type. A phase compensation function is defined as follows:

(11) $$H_{m}\left(\omega ,p\right)=w_{m}e^{j\omega {\hat{\tau }}_{m}\left(p\right)}$$

where $w_{m}$ denotes the channel weight, and ${\hat{\tau }}_{m}(p)$ is the expected time delay calculated based on the trial position p. Superposing the compensated signals of all channels, the array output is obtained as follows:

(12) $$Y\left(\omega ,p\right)=\sum_{m=1}^{M}H_{m}\left(\omega ,p\right)X_{m}\left(\omega \right)=S\left(\omega \right)\sum_{m=1}^{M}w_{m}G_{m}\left(\omega ,p_{s}\right)e^{j\omega {\hat{\tau }}_{m}\left(p\right)}+\tilde{N}\left(\omega \right)$$

When the trial position coincides with the real sound source position ($p=p_{s}$)), if the difference in amplitude attenuation is neglected, the phase compensation term $e^{j\omega {\hat{\tau }}_{m}(p)}$ exactly cancels out the propagation phase delay $e^{-j\omega \tau_{m}}$. The signals of all channels thus achieve in-phase superposition, and the amplitude of the array output reaches its maximum value:

(13) $$\left|Y\left(\omega ,p_{s}\right)\right|\approx \left|S\left(\omega \right)\right|\left|\sum_{m=1}^{M}w_{m}\right|$$

Sound source localization based on beamforming can be formulated as a maximum likelihood estimation problem. The spatial spectrum function is defined as follows:

(14) $$P\left(p\right)=\int_{\Omega }|Y\left(\omega ,p\right){|}^{2}d\omega =\int_{\Omega }{\left|\sum_{m=1}^{M}w_{m}X_{m}\left(\omega \right)e^{j\omega {\hat{\tau }}_{m}\left(p\right)}\right|}^{2}d\omega$$

where Ω denotes the effective frequency band range. The maximum likelihood estimation of the sound source position is obtained by maximizing the spatial spectrum function:

(15) $${\hat{p}}_{s}=arg\underset{p\in P}{max}P\left(p\right)$$

The spatial spectrum P(p) corresponds to the negative log-likelihood function under Gaussian noise assumption. Maximizing P(p) is equivalent to minimizing the residual between observed and predicted signals.

In the considered near-field region, P(p) is continuous over the search domain. When ground reflection is properly compensated and multi-frequency incoherent averaging is applied, the spatial spectrum exhibits a single dominant peak at the true source position, as verified by the experimental results in Section 4. Without reflection compensation, reflection-induced spurious peaks can appear. A coarse-to-fine grid search is therefore employed to locate the global maximum. Under the assumption of zero-mean noise uncorrelated with the source, the discrete-frequency approximation of Equation (14) reduces to Equation (16).

(16) $$P\left(p\right)\approx \sum_{k=1}^{K}{\left|\sum_{m=1}^{M}w_{m}X_{m}\left(\omega_{k}\right)exp\left[j\omega_{k}{\hat{\tau }}_{m}\left(p\right)\right]\right|}^{2}$$

where $\omega_{k}$ denotes the K discrete-frequency points.

In industrial environments, colored noise with spatial covariance $R_{n}(\omega )$ may render $P_{noise}(p)$ position-dependent and bias the spectral peak. An optional pre-whitening stage ($\tilde{X}=R_{n}^{-1/2}X$) is therefore provided, with $R_{n}$ estimated during a source-silent interval. The present experiments operate under approximately white ambient noise over 500 Hz–3 kHz, justifying the simplified form.

In addition, to improve computational efficiency, the system adopts a hierarchical search strategy: First, a global search is performed on a coarse grid (Δ=0.02 m), followed by a refined search on a fine grid (Δ=0.005 m) near the extreme point. Meanwhile, by utilizing the symmetry of ground reflection, ${\hat{\tau }}_{m}(p)$ is precomputed and stored to reduce the real-time computational load.

While the introduction of the ground-reflection model increases the computational complexity, it significantly improves the localization accuracy, and is particularly suitable for the near-field region where $|p_{s}-p_{m}|<2D^{2}/\lambda$ (where *D* denotes the array aperture and λ represents the wavelength). Experiments show that the system can meet the real-time processing requirements within a search range of 0.5 m × 0.5 m.

### 2.3. Spatial Aliasing Processing and Microphone Spacing Selection

In the signal processing of microphone arrays, the relationship between the array element spacing d and the wavelength λ abides by the spatial sampling theory. According to the spatial Nyquist criterion, to avoid spatial aliasing, the array element spacing must satisfy the following condition:

(17) $$D\leq \frac{\lambda_{min}}{2}=\frac{c}{2f_{max}}$$

where $\lambda_{min}$ denotes the minimum wavelength and $f_{max}$ represents the maximum frequency. For the system in this paper, with $f_{max}=3000\;Hz$ and c=343 m/s, the theoretical minimum spacing is calculated as

(18) $$D_{min}=\frac{343}{2\times 3000}\approx 0.0571\;m=57.1\;mm$$

However, the spacing adopted in the practical system is d=0.1 m=100 mm, which is larger than $D_{min}$, and this will lead to spatial aliasing in the high-frequency components.

When d>λ/2, the spatial frequency u=sinθ/λ exceeds the Nyquist frequency 1/(2d), and the phase difference $\phi_{m}=2\pi d_{m}sin\theta /\lambda$ exhibits ambiguity by an integer multiple of 2π. For a linear array, the steering vector a(θ,f) exhibits periodicity:

(19) $$A\left(\theta +arcsin\left(\frac{n\lambda }{d}\right),f\right)=a\left(\theta ,f\right),\;n\in Z$$

In other words, for far-field plane-wave incidence, two directions separated by $arcsin\left(\frac{n\lambda }{d}\right)$ produce identical steering vectors, leading to direction-of-arrival ambiguity; for the array with d=0.1 m, the aliasing frequency threshold is as follows:

(20) $$F_{alias}=\frac{c}{2d}=\frac{343}{0.2}=1715\;Hz$$

This means that signals with a frequency higher than 1715 Hz will exhibit spatial aliasing.

Under the near-field condition ($r<2D^{2}/\lambda$, where D is the array aperture), acoustic waves propagate in the form of spherical waves, and the phase difference is related not only to the direction but also to the distance:

(21) $$\Phi_{m}\left(p_{s}\right)=\frac{2\pi }{\lambda }\left(∥p_{s}-p_{m}∥-∥p_{s}-p_{ref}∥\right)$$

For aliasing to produce an ambiguous position $p^{\prime }$, all M microphones must satisfy $∥p-p_{m}∥-∥p-p_{ref}∥=∥p^{\prime }-p_{m}∥-∥p^{\prime }-p_{ref}∥+n\lambda$ simultaneously. In the far field, the range difference linearizes to a direction-only term, recovering the periodic ambiguity of Equation (19). In the near field, the quadratic curvature term $∥p_{m}-p_{ref}{∥}^{2}/(2r)$ is non-negligible. When the number of array elements satisfies *M* ≥ 3, the system of equations becomes overdetermined, and no solution with $p^{\prime }\neq p$ can satisfy all equations. Hence, near-field spherical-wave propagation inherently suppresses spatial aliasing. For the present array, r≤0.35 m ≪$2D^{2}/\lambda \approx 1.57\;m$, confirming a comfortable margin. For the system in this paper, with a search range *r* = 0.35 m (safety margin), an array aperture *D* = 0.3 m and a maximum frequency f=3000 Hz:

(22) $$\frac{2D^{2}}{\lambda }=\frac{2\times 0.3^{2}}{\frac{343}{3000}}\approx 1.574\;m>r$$

This satisfies the near-field condition, thus effectively avoiding spatial aliasing.

Meanwhile, by utilizing incoherent frequency averaging of the wideband signal, the spatial spectrum is defined as the sum of energies at multiple frequency points:

(23) $$P_{WB}\left(p\right)=\frac{1}{K}\sum_{k=1}^{K}P\left(p,f_{k}\right)$$

The spurious peaks induced by aliasing have different spatial positions at different frequencies, while the real sound source position remains consistent across all frequencies. Through multi-frequency averaging, the spurious peaks are suppressed:

(24) $$E\left[P_{alias}\left(p\right)\right]\approx \frac{1}{K}\sum_{k}\delta \left(p-p_{alias,k}\right)\to 0$$

In Equation (24), $p_{alias,k}$ denotes the position vector of the aliasing-induced spurious peak at frequency $f_{k}$, expressed in the same coordinate system as the trial position *p*. As discussed above, $p_{alias,k}$ varies across frequency bins while the true source position remains fixed. A frequency weighting function w(f) is constructed to reduce the weight of frequency bands with severe aliasing:

(25) $$\begin{array}{r} W\left(f\right)=\left\{\begin{array}{ll} 1, & f\leq f_{alias}\\ exp\left(-\frac{(f-f_{alias}{)}^{2}}{2\sigma_{f}^{2}}\right), & f>f_{alias} \end{array}\right. \end{array}$$

where $\sigma_{f}$ controls the decay rate of the weighting function; $\sigma_{f}$ = 500 Hz is used herein, gradually suppressing frequency bands above $f_{alias}$; the Gaussian roll-off in Equation (25) was designed to avoid the drawbacks of two simpler weighting strategies. A hard cutoff at $f_{alias}$ discards all high-frequency information abruptly, reducing the effective bandwidth available for localization. Uniform weighting (*w(f)* = 1) retains the aliased bands at full strength, leaving residual spurious peaks in the averaged spectrum. The Gaussian roll-off progressively attenuates bands above $f_{alias}$ rather than discarding them, retaining partially usable high-frequency content while suppressing the most severely aliased bins. The weighted spatial spectrum is given by

(26) $$P_{w}\left(p\right)=\sum_{k=1}^{K}w\left(f_{k}\right)P\left(p,f_{k}\right)$$

After incorporating aliasing suppression, the optimized localization function is given by

(27) $${\;\hat{p}}_{s}=arg\underset{p}{max}\sum_{k=1}^{K}w\left(f_{k}\right){\left|\sum_{m=1}^{M}X_{m}\left(f_{k}\right)exp\left[j2\pi f_{k}{\hat{\tau }}_{m}\left(p\right)\right]\right|}^{2}$$

Here ${\hat{\tau }}_{m}(p)$ denotes the effective phase delay derived from the total Green’s function in Equation (8). In implementation, the beamforming weight uses the complex conjugate of $G_{m}(\omega ,p_{s})$ from Equation (10) to jointly match both the direct and reflected path phases.

## 3. System Implementation

**Figure 3:**
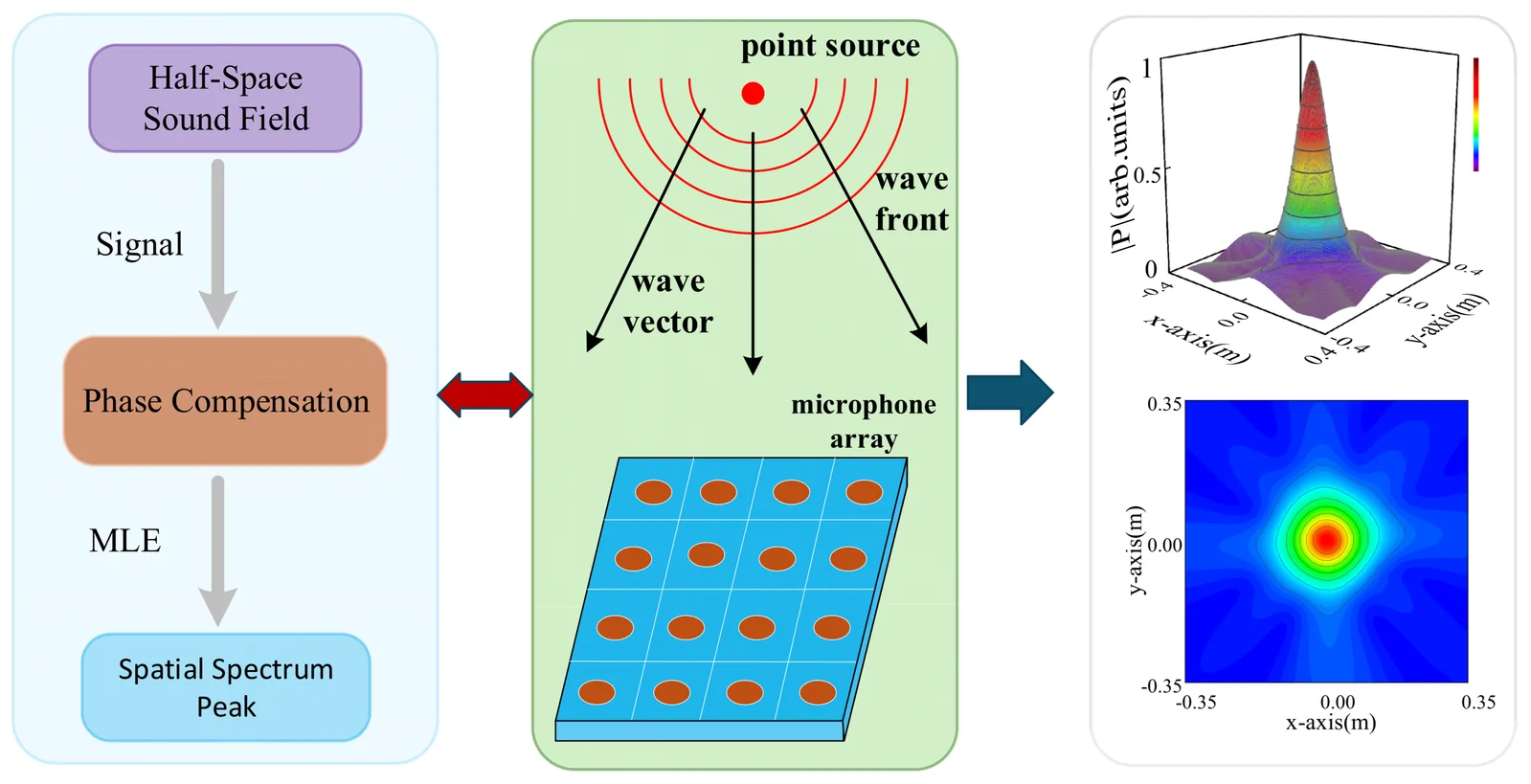
Schematic of beam scanning algorithm based on phase compensation and simulation results.

For the acoustic localization algorithm based on the MEMS array, a sound source localization system with a 4 × 4 microphone array is designed, as shown in Figure 3. The experimental equipment of the system consists of a point sound source, a microphone array board, a computer, and a self-developed sound source localization program based on MATLAB 2025b software. The sound source is a calibrated full-range loudspeaker (HiVi M3N, Zhuhai HiVi Acoustics Technology Co., Ltd., Zhuhai, China) driven by broadband white noise (200 Hz–3 kHz, 80 dB SPL at 0.2 m). The 4 × 4 MEMS array (SSP-MA16-U2, assembled with MEMS microphones from Goertek Inc., Weifang, Shandong, China; hardware detailed in Appendix A) is mounted with its plane at *z* = 0 and the ground plane at *z* = −10 mm. Prior to each session, microphone gains are normalized using a 1 kHz reference tone, and α is calibrated by matching the measured transfer function between the source and a reference microphone to the free-field prediction, yielding α ≈ 0.95 for the concrete floor. The system adopts a modular design concept, including four core modules: the Data Reception Module, the Signal Preprocessing Module, the Analysis Module, and the Localization Result Output Module. The specific workflow is as follows. (a) Data Reception: the 4 × 4 MEMS array (16 elements, d=0.1 m) samples synchronously at 48 kHz, 16-bit, via USB interface to a Raspberry Pi 4B (Raspberry Pi Ltd., Cambridge, UK), which buffers and streams the multi-channel data. (b) Signal Preprocessing: each channel is framed (*N* = 1024, 21 ms, 50% overlap, Hanning window), DC-corrected, and high-pass filtered (4th-order Butterworth, $f_{c}=200\;Hz$). A 1024-point FFT (Δf≈47 Hz) retains bins within 200 Hz–3 kHz. (c) Analysis: for each grid candidate, Green’s-function delays from Equation (8) are computed, phase compensation is applied, and the wideband spectrum is accumulated via Equations (23)–(26); position is refined by coarse-to-fine grid search ($\Delta_{coarse}=0.02\;m$, $\Delta_{fine}=0.005\;m$). (d) Output: estimated coordinates refresh every 0.5 s in automatic broadband or manual single-frequency mode. Based on the near-field spherical-wave model and the Green’s function incorporating ground reflection, the Analysis Module calculates the theoretical time delay for each trial position in the preset search grid, and achieves in-phase superposition of the signals of each channel under the assumption of this position through phase compensation to generate a beam output. A wideband processing strategy is adopted to fuse the spatial spectra of multiple prominent frequency points; following the maximum likelihood estimation criterion, the grid point that maximizes the beamforming output energy is identified as the sound source position estimation. Finally, the Localization Result Output Module displays the localization results in a visual form on the interactive interface in real time, and supports refined analysis for specific frequencies in manual mode. The entire workflow is executed cyclically with a period of 0.5 s to realize real-time tracking and localization of dynamic sound sources. The microphone array adopts a square array layout with an element spacing of 0.1 m, and the array plane is set at *z* = 0. The operating distance of the sound source is *z* ≈ 0.1 m–0.3 m, the ground height is set to *z* = −0.01 m (below the array), and the reflection coefficient is α = 0.95 (supports customized modification based on the material of the reflecting surface).

The purpose of the simulation experiment is to verify the performance of the localization system by evaluating the sound source localization effect achieved from simulating actual sound source propagation using a dataset. The dataset simulates the process in which a point sound source, located directly above the microphone array at the coordinate (0,0), emits acoustic signals omnidirectionally; the signals are then collected by the microphone array and imported into the localization system. After undergoing signal preprocessing, the collected data is processed by the acoustic localization algorithm, and the simulation results are obtained.

The results in the figure show the 2D contour and 3D view of the simulated sound pressure field on the horizontal observation plane (X, Y coordinates, unit: m) of the point sound source localization system under a free-field environment. Color mapping is adopted to quantitatively characterize the spatial distribution characteristics of the sound pressure amplitude (unit: Pa). It can be seen from the figure that the sound pressure in the observation plane exhibits significant spatial inhomogeneity, forming a focal region of extreme sound pressure in the central area of the X and Y coordinates with the peak sound pressure reaching 0.6020 Pa. As the distance between the spatial position and this focal region increases, the sound pressure shows a continuous monotonic attenuation trend, dropping to a background level close to 0 Pa in the edge region of the observation plane. This simulation result is highly consistent with the acoustic radiation law of a point sound source in the free field of a lossless medium, and quantitatively reflects the spatial inhomogeneity of the sound pressure.

## 4. Experimental Results and Discussion

Real sound sources are employed to conduct tests on the sound source localization system, and the corresponding experimental results are obtained.

**Figure 4:**
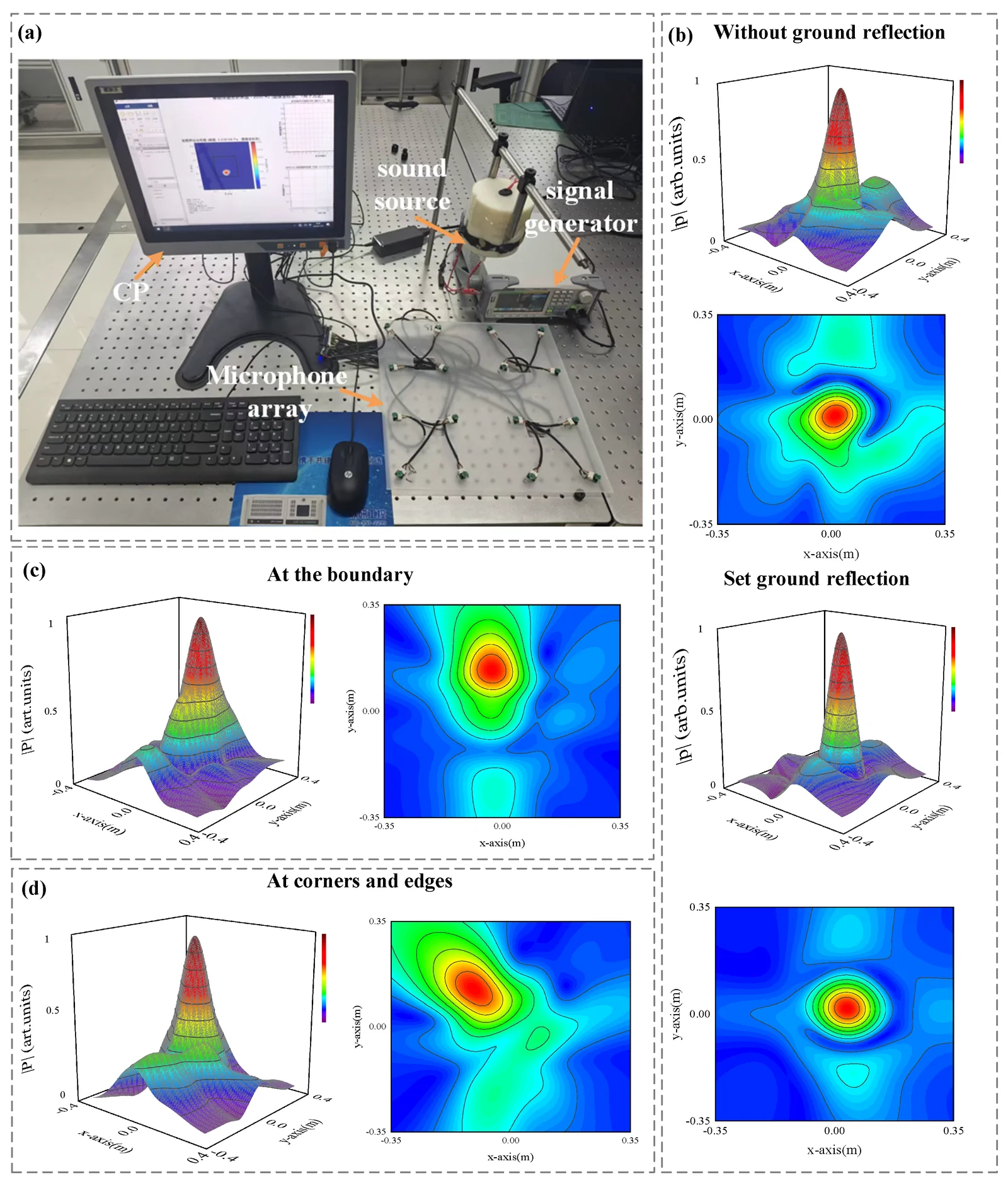
Experimental setup and localization results. (**a**) Photograph: 4 × 4 MEMS array (*d* = 0.1 m, *D* = 0.3 m) on concrete floor (*α* = 0.95), point source at (0, 0, 0.2 m). (**b**) Sound pressure field and 2D contour maps. Without ground-reflection compensation (RMSE = 4.2 cm, MSPR = 10.5 dB). With compensation (α = 0.95, RMSE = 0.9 cm, MSPR = 19.3 dB). *f* = 200 Hz–3 kHz. True source position marked by white cross. (**c**) Localization result at boundary position (0, 0.18, 0.2 m). (**d**) Localization result at corner position (−0.18, 0.18, 0.2 m). Both have ground-reflection compensation (α = 0.95).

A real point sound source is placed directly above the microphone array at the coordinates (0, 0) with a vertical height of 0.2 m (a representative height in the 0.1–0.3 m working range) above the array plane. The point sound source emits acoustic signals, which are then collected by the microphone array directly below and sent to the system for subsequent processing, as shown in Figure 4a.

The results in Figure 4b represent the 3D distribution of the sound pressure field and the corresponding 2D contour maps on the horizontal observation plane (X, Y coordinates, unit: m) of the point sound source localization system after actual tests. To quantitatively evaluate the influence of environmental multipath effects on localization accuracy, two sets of comparative experiments were conducted: one without ground-reflection correction and the other with ground-reflection correction based on the image-source method. The uncorrected case is equivalent to conventional near-field beamforming using the free-space Green’s function, as it retains only the direct-path term in Equation (10) without the reflected component; the corrected case represents the proposed method.

To quantitatively evaluate the performance of the proposed algorithm, two standard evaluation metrics are adopted: Root Mean Square Error (RMSE) [30] for localization accuracy, and main-to-sidelobe peak ratio (MSPR) [31] for anti-interference ability, defined as

(28) $$RMSE=\sqrt{\frac{1}{N}\sum_{i=1}^{N}\left[(x_{i}-{\hat{x}}_{i}{)}^{2}+(y_{i}-{\hat{y}}_{i}{)}^{2}\right]}$$

where N is the number of test points, and $\left(x_{i},y_{i}\right)$ and $\left({\hat{x}}_{i},{\hat{y}}_{i}\right)$ are the true and estimated coordinates, respectively, and

(29) $$MSPR=20{log}_{10}\left(\frac{P_{main}}{P_{max\_sidelobe}}\right)\;\left(\mathrm{dB}\right)$$

where $P_{main}$ is the main peak amplitude and $P_{max\_sidelobe}$ is the maximum sidelobe amplitude including reflection-induced spurious peaks.

Without ground-reflection correction, the average RMSE is 4.2 cm and the MSPR is only 10.5 dB, showing a multi-peak distribution with a 4 cm main peak shift and distinct spurious peaks accounting for 30% of the main peak amplitude; after applying the proposed correction algorithm, the average RMSE is reduced to 0.9 cm (<0.015 m) and the MSPR is significantly improved to 19.3 dB, with the main peak accurately returning to (0, 0); spurious peaks are suppressed, and the sound energy concentration improves by approximately 40%. Figure 4c shows the localization result at a boundary position (0, 0.18, 0.2 m). With compensation, the main peak remains at the true source position and the MSPR stays above 18.5 dB, confirming that the reflection model holds at the edges of the search region. Figure 4d shows results at a corner position (−0.18, 0.18, 0.2 m). The compensated localization maintains an RMSE below 0.015 m, demonstrating that the method remains effective under the combined influence of larger incident angle and ground reflection at the search region corner. Additional tests at different heights within 0.1–0.3 m confirm that the average RMSE remains consistently below 0.015 m, and the average single-frame processing time (SFPT) of the system is 0.45 s, fully meeting the real-time requirement of 0.5 s per frame.

The 4 cm peak shift observed without compensation is consistent with the phase mismatch introduced by the unmodeled reflected path: when the free-field Green’s function is used, the reflected arrival contributes a frequency-dependent bias that displaces the spatial spectrum maximum from the true source position. After compensation, the main peak returns to (0, 0) and the MSPR improves from 10.5 dB to 19.3 dB, confirming that embedding the reflected path into the beamforming kernel Equations (7) and (8) enables the reflected energy to contribute constructively rather than as coherent interference. This result is consistent with the matched-field processing principle: incorporating propagation physics into the replica vector improves localization accuracy and sidelobe suppression. From this perspective, the proposed method can be understood as near-field matched-field processing in which the environmental model consists of a single image-source path parameterized by α. This distinguishes the method from two extremes. At one end, free-field beamforming uses no environmental model and treats all reflections as unmodeled interference, resulting in the 4.2 cm RMSE of the uncompensated baseline. At the other end, full-field matched-field processing as practiced in ocean acoustics requires detailed knowledge of sound speed profiles, bathymetry, and seabed properties, and solves the wave equation numerically at each grid point, which is computationally prohibitive for real-time operation. The proposed image-source model occupies a practical middle ground: it captures the dominant coherent reflection with one calibrated parameter while retaining the computational simplicity of frequency-domain beamforming, enabling real-time operation on compact hardware. In terms of computational performance, the per-frame complexity is comparable to conventional delay-and-sum beamforming and substantially lower than in pairwise cross-correlation methods such as SRP-PHAT. The measured SFPT of 0.45 s meets the 0.5 s real-time target; The per-frame cost scales as O (K · M · Ntotal); doubling M or quadrupling Ntotal would increase processing time by factors of approximately 2 and 4, respectively. Real-time operation can be preserved through bandwidth reduction, grid coarsening, or a compiled-language implementation. Extension to multiple sources via multi-peak detection is conceptually feasible but increases computational demands and is reserved for future work. Porting the MATLAB implementation to a compiled language is expected to further reduce processing time. Compared with reported near-field beamforming results under free-field conditions, the achieved RMSE of 0.9 cm with compensation is within the accuracy range of existing systems [32,33], with the additional benefit that this accuracy is maintained in the presence of a reflective ground surface. Table 1 situates the present results within the context of published near-field localization studies. Although differences in array configuration, source distance, and acoustic environment preclude a strictly controlled comparison, the table provides a useful reference for the performance range achievable with current methods. The proposed approach achieves accuracy competitive with or exceeding that of existing systems while uniquely operating under a reflective ground condition. The benchmark entries differ in array geometry and environment from the present study; they serve as representative performance references rather than strictly controlled comparisons. The MUSIC-family entry reports simulation results for classical spherical-harmonics MUSIC; sub-centimeter accuracy (0.49 cm) is achieved only at 40 dB SNR, degrading to 28.91 cm at 30 dB and 49.5 cm at 10–20 dB, illustrating the SNR sensitivity that motivated the broadband design of the proposed method [34]. The SRP-PHAT entry reports the average localization error over randomly placed sources in a room-scale environment (RT = 0.1 s, SNR 10–20 dB); the source distance was not fixed [35].

Quantitatively, the achieved RMSE of 0.9 cm is lower than all experimental benchmarks in Table 1 by a factor of 3 to 32. The only exception is the 0.49 cm MUSIC simulation best-case, obtained at 40 dB SNR under idealized free-field conditions without a reflective surface. The comparative experiment in Figure 4b isolates the contribution of the compensation: on the same hardware and under the same experimental conditions, the configuration with the reflected path embedded in the beamforming kernel reduces the RMSE from 4.2 cm to 0.9 cm, a 78% reduction attributable to the proposed compensation.

The image-source model assumes a flat, homogeneous, specularly reflecting ground. In practice, industrial floors combine concrete, grating, and oil patches with spatially varying impedance; indoor walls contribute unmodeled multipath; outdoor terrains such as gravel, grass, and slopes introduce frequency-dependent absorption and diffuse scattering. These non-idealities introduce bias into the compensation when the assumed reflection coefficient α deviates from its true value. Mitigation strategies, including on-site α calibration, band-limited operation, and phase mismatch masking, are reserved for deployment-specific validation.

## 5. Conclusions

This paper has proposed a real-time sound source localization method that embeds image-source ground-reflection compensation directly into the near-field beamforming Green’s function. A real-time localization system based on a 4 × 4 MEMS array was designed and experimentally validated. Experimental results demonstrate that the system can achieve high-precision real-time localization of sound sources, and simultaneously realize significant suppression of concomitant sidelobe interference. Consequently, the corresponding spatial coordinates are accurately matched with the actual positions of sound sources without any offset caused by interference, demonstrating its engineering applicability. Several limitations should be noted. The current system assumes a single point source and has been validated within a working distance of 0.1–0.3 m. Beyond 0.3 m, two effects degrade performance. First, as the source moves toward the far-field threshold $\frac{2D^{2}}{\lambda }$ ≈ 1.57 m, the wavefront curvature decreases, reducing the phase diversity that enables unambiguous near-field 2D localization. Second, the path-length difference between the direct and reflected rays decreases relative to the wavelength, making the reflection-induced phase shift progressively smaller and harder to distinguish from measurement noise. Conversely, below 0.1 m, the Fresnel approximation underlying the image-source model weakens and microphone near-field effects become non-negligible. These physical constraints define the operational range of the current system; extending this range would require a larger array aperture or a hybrid near-field/far-field processing strategy. The image-source model requires a known reflection coefficient and flat homogeneous ground; non-ideal surfaces introduce bias. Spatial aliasing above 1715 Hz, caused by the 0.1 m array spacing, is mitigated but not eliminated by frequency weighting. Finally, performance in strongly reverberant environments remains to be evaluated. Additionally, the present experiments were conducted in a single acoustic environment with a concrete floor and low reverberation. Validation under diverse surface materials, elevated background noise, and stronger reverberation is needed to establish the generality of the approach. It provides an effective solution for near-field sound source localization in industrial scenarios such as equipment fault diagnosis and acoustic monitoring. A systematic comparison with other localization frameworks such as TDOA-based methods under the same reflection-compensated configuration is warranted in future work. An in-house experimental implementation of MUSIC and SRP-PHAT on the same hardware and dataset is a high-priority direction for follow-up work. Extension to multiple simultaneous sources is also possible in principle, as the spatial spectrum P(p) can exhibit local maxima at each source position. Practical multi-source localization would require a peak-separation criterion, higher grid resolution to resolve closely spaced sources, and handling of cross-term interference between simultaneously active sources. These extensions, together with 3D super-resolution localization and strong reverberation suppression, are left for future work.

## Figures and Tables

**Table 1:** Reported performance of near-field sound source localization methods.

Method	Array	Search Region (m)	RMSE (cm)	Reflective Surface	Ref.
TDOA-EFPI localization	3-mic linear	0.8 × 0.6	3.13	No	[36]
PSO-optimized TDOA	7-mic 3D-optimized	5–15	Not reported	No	[37]
Ground-reflection-compensated	16-MEMS planar	0.5 × 0.5	0.9	Concrete ground	This work
Near-field MUSIC	Spherical array	0.1–0.8	0.49 (40 dB)	No	[34]
Weighted SRP-PHAT	24-mic (L-shaped)	Room-scale	13.7	No	[35]

## Data Availability

The raw data supporting the conclusions of this article will be made available by the authors on request.

## Nomenclature

**Symbol**	**Description**	**Unit**
p(r,t)	Time-domain sound pressure	Pa
P(r,ω)	Frequency-domain sound pressure	Pa·s
c	Speed of sound (343 m/s)	m/s
k	Wavenumber (=ω/c)	rad/m
ω	Angular frequency	rad/s
f	Acoustic frequency	Hz
$G(r,r_{0},\omega )$	Free-space Green’s function	m^−1^
$G_{total}$	Total Green’s function with ground reflection	m^−1^
α	Ground-reflection coefficient	—
$r_{0}$	True source position $\left(x_{0},y_{0},z_{0}\right)$	m
$r_{0^{\prime }}$	Image-source position $\left(x_{0},y_{0},-z_{0}\right)$	m
r	Observation point	m
$p_{m}$	Position of m-th microphone	m
M	Number of microphones	—
d	Microphone spacing	m
D	Array aperture	m
λ	Wavelength	m
$X_{m}(\omega )$	Frequency-domain signal at m-th microphone	Pa·s
$N_{m}(\omega )$	Noise at m-th microphone	Pa·s
$w_{m}$	Channel weight	—
$\tau_{m}(p)$	Propagation delay from source candidate p to m-th microphone	s
P(p)	Spatial spectrum at candidate position p	—
$p_{s}$	Estimated source position	m
$f_{s}$	Sampling rate	Hz
N	Frame length	samples
Δt	Frame interval	s
$\sigma_{f}$	Decay rate of frequency weighting function	Hz

## Appendix A. Instrumentation Specifications

**Component**	**Model**	**Key Specifications**
USB MEMS array	SSP-MA16-U2	16 MEMS microphones arranged in a 4 × 4 grid, element spacing d = 0.1 m, 16-bit quantization, 48 kHz sampling rate, USB 2.0 interface
Processing unit	Raspberry Pi 4B	4-core Cortex-A72 processor, 8 GB RAM
Array frame	Custom aluminum	Array aperture D = 0.3 m, 10 mm ground clearance
